# The Modifiability of the Stereotype Content Model Toward Older Adult Men and Women

**DOI:** 10.1093/geroni/igaa057.1884

**Published:** 2020-12-16

**Authors:** Michael Vale Toni Bisconti, Jennifer Sublett

**Affiliations:** University of Akron, Akron, Ohio, United States

## Abstract

Older adults are often stereotyped in a paternalistic manner (warm, but incompetent), deserving of assistance regardless of their need. We have examined the veracity and malleability of this paternalistic stereotype using an experimental vignette with both male and female targets. Younger adults (N = 717) deemed it more necessary and appropriate to offer unnecessary help to older adults in a grocery store scenario. Additionally, competence was malleable for both older adult male and female targets if the older adults denied the offer of help. Interestingly, older women were viewed as warm, which did not change as a function of their response, whereas older men were initially viewed as colder, but their warmth ratings increased. In light of these findings, we will discuss the intersection of age and gender when considering the malleability of the warmth and competence dimensions of the paternalistic older adult stereotype.

